# Association of IL-17 inhibitor therapy with glycemic, lipid profiles, and systemic inflammation in psoriasis patients with metabolic comorbidities: a retrospective cohort study

**DOI:** 10.3389/fmed.2026.1891303

**Published:** 2026-07-29

**Authors:** Xianzhe Zhang, Man Zhang, Zhenhua Nie, Yan Xu, Haiyan Xu, YunYun Li, Jun Li, Mengyuan Zhu, Junying Li

**Affiliations:** 1 Department of Dermatology, Tianjin Academy of Traditional Chinese Medicine Affiliated Hospital, Tianjin, China; 2 Tianjin University of Traditional Chinese Medicine, Tianjin, China

**Keywords:** IL-17 inhibitor, metabolic comorbidities, psoriasis, systemic inflammation, systemic inflammation response index

## Abstract

**Background:**

Moderate-to-severe plaque psoriasis frequently coexists with metabolic comorbidities, yet the impact of interleukin-17 (IL-17) inhibitors on metabolic parameters remains inadequately characterized. This study evaluated the effects of IL-17 inhibitor therapy on glycemic control, lipid profiles, uric acid, and novel systemic inflammatory indices in psoriasis patients with metabolic comorbidities.

**Objective:**

This 48-week single-center retrospective study included 30 patients with moderate-to-severe plaque psoriasis (Psoriasis Area and Severity Index [PASI] ≥10 with ≥1 metabolic comorbidity) receiving IL-17 inhibitors (June 2023-December 2024). Fasting glucose, lipid profiles, uric acid, serum β2-microglobulin, urinary albumin-to-creatinine ratio (UACR), homocysteine, systemic inflammation response index (SIRI), and systemic immune-inflammation index (SII) were assessed at baseline and weeks 12, 24, 36, and 48. The primary outcome was fasting glucose reduction ≥10% at week 48.

**Results:**

After 48 weeks of treatment, PASI score decreased from 18.6 ± 5.4 to 2.8 ± 1.2 (*P* < 0.001). Fasting glucose decreased from 6.2 ± 1.4 to 5.3 ± 0.9 mmol/L (*P* < 0.001), triglycerides from 2.1 ± 0.8 to 1.6 ± 0.5 mmol/L (*P* = 0.002), uric acid from 435 ± 52 to 378 ± 41 μmol/L (*P* < 0.001), and homocysteine from 14.2 ± 3.5 to 10.8 ± 2.6 μmol/L (P < 0.001). SIRI decreased from 1.85 ± 0.62 to 0.92 ± 0.31 (*P* < 0.001), and SII from 680 ± 145 to 420 ± 98 (*P* < 0.001). Multivariate analysis suggested that SIRI reduction may be an independent predictor of glycemic improvement [odds ratio (OR) = 2.68, 95% confidence interval (CI): 1.21–5.93, *P* = 0.015] in this cohort.

**Conclusion:**

This study suggests that IL-17 inhibitor therapy is associated with improvements in glycemic control, triglycerides, uric acid, homocysteine, and systemic inflammation in psoriasis patients with metabolic comorbidities. Preliminary evidence indicates that SIRI may serve as a practical clinical indicator for assessing metabolic improvement, although prospective validation is required. However, given the observational design, small sample size, lack of a control group, and absence of systematic data on lifestyle factors and direct measures of insulin resistance, these findings should be interpreted as hypothesis-generating and do not establish causality. Confirmation in larger prospective controlled studies is needed before any clinical recommendations can be made.

## 1 Introduction

Psoriasis affects 2–3% of the global population, with moderate-to-severe cases comprising 20–30% (1). Beyond the substantial cutaneous burden, patients with psoriasis face significantly increased risks of metabolic comorbidities. Epidemiological evidence demonstrates that individuals with psoriasis have a 30–40% higher risk of developing type 2 diabetes mellitus, a 20–30% increased risk of dyslipidemia, and a 40–50% elevated risk of obesity compared with the general population (2). The shared inflammatory pathways, particularly the interleukin-23 (IL-23)/T helper 17 (Th17) axis, underpin both psoriatic skin inflammation and systemic metabolic disturbances, establishing a paradigm of “psoriatic disease” that extends beyond dermatological manifestations (3). This interconnection suggests that effective control of systemic inflammation may yield concomitant metabolic benefits, yet the magnitude and consistency of such effects across different biologic classes remain incompletely defined.

The IL-23/Th17 signaling cascade plays a central role in psoriasis pathogenesis, with IL-17 serving as a key effector cytokine driving keratinocyte hyperproliferation and recruitment of inflammatory cells into the skin (4). IL-17 inhibitors, including secukinumab and ixekizumab, have demonstrated remarkable efficacy in moderate-to-severe psoriasis, with phase III trials reporting PASI 90 response rates of 70–80% at 12 weeks (5). These agents selectively neutralize IL-17A, thereby blocking downstream inflammatory cascades that contribute to both cutaneous and systemic inflammation. However, the impact of IL-17 blockade on metabolic parameters in psoriasis patients remains incompletely understood. Preclinical investigations have implicated IL-17 in adipose tissue inflammation, insulin resistance, and hepatic lipid metabolism, suggesting that therapeutic targeting of this pathway might confer metabolic benefits (6). Nevertheless, existing clinical studies are predominantly small-scale or cross-sectional, lacking systematic longitudinal assessment of metabolic outcomes over extended follow-up periods.

Emerging evidence highlights the utility of novel composite inflammatory indices derived from routine complete blood counts as surrogate markers of systemic inflammation (7). The SIRI, calculated as neutrophil count × monocyte count / lymphocyte count, and the SII, calculated as neutrophil count × platelet count / lymphocyte count, integrate information from multiple leukocyte lineages to provide a more comprehensive assessment of inflammatory burden compared with individual parameters (8). NLR (neutrophil-to-lymphocyte ratio) and PLR (platelet-to-lymphocyte ratio) are simpler inflammatory indices associated with cardiovascular disease, diabetes, and metabolic syndrome (9, 10). Unlike CRP (C-reactive protein), they are inexpensive, widely available, and not subject to circadian variation. However, their changes during biologic therapy for psoriasis and association with metabolic improvement remain uninvestigated.

Current knowledge gaps regarding IL-17 inhibitor effects on metabolic comorbidities in psoriasis include several critical issues. First, most studies focus on isolated metabolic parameters such as fasting glucose, without comprehensive evaluation of lipid profiles, uric acid, renal function-related indices, or novel inflammatory markers (11). Second, relatively short follow-up durations (typically 12–24 weeks) limit assessment of sustained treatment effects and long-term metabolic trajectories (12, 13). Third, the relationship between inflammatory marker reduction and metabolic improvement remains inadequately characterized, with few studies examining whether the degree of inflammation attenuation predicts the magnitude of metabolic benefit. Fourth, the potential differential effects of IL-17 inhibitors on various lipid subfractions (triglycerides vs. cholesterol) have not been adequately explored. As biologic therapies become increasingly prevalent in psoriasis management, clarifying their impact on metabolic comorbidities carries substantial clinical importance for optimizing treatment selection and comprehensive patient care.

This study was designed to systematically evaluate the dynamic changes in glycemic control, lipid profiles, uric acid, serum β2-microglobulin, UACR, homocysteine, SIRI, and SII during 48 weeks of IL-17 inhibitor therapy in patients with moderate-to-severe plaque psoriasis and metabolic comorbidities. We hypothesized that IL-17 inhibitor treatment would improve both cutaneous disease activity and metabolic parameters through attenuation of systemic inflammation, and that SIRI and SII would serve as practical clinical indicators for assessing metabolic improvement. The primary objective was to determine the proportion of patients achieving clinically meaningful glycemic improvement (≥10% reduction in fasting glucose) at week 48 and to explore potential predictors of this response in an exploratory manner. Secondary objectives included characterizing the temporal patterns of metabolic and inflammatory changes and examining correlations between cutaneous response and metabolic outcomes. The findings may provide novel evidence supporting the pleiotropic benefits of IL-17 inhibitors in psoriatic disease management and inform clinical decision-making regarding biologic selection in patients with metabolic comorbidities.

## 2 Materials and methods

### 2.1 Study design and ethical approval

This single-center retrospective cohort study at Tianjin Academy of Traditional Chinese Medicine Affiliated Hospital Unit received IRB approval, adhered to Declaration of Helsinki principles, and followed STROBE (Strengthening the Reporting of Observational Studies in Epidemiology) guidelines. Informed consent was waived; data were anonymized.

### 2.2 Study population and eligibility criteria

We screened electronic health records of all adult patients (age ≥18 years) with confirmed moderate-to-severe plaque psoriasis who initiated IL-17 inhibitor therapy at the dermatology clinic of XXX Unit between June 1, 2023, and December 31, 2024. Inclusion criteria were: (1) moderate-to-severe plaque psoriasis defined by PASI score ≥10; (2) presence of at least one metabolic comorbidity, including obesity [body mass index (BMI) ≥28 kg/m^2^], dysglycemia (fasting glucose ≥6.1 mmol/L or established diagnosis of type 2 diabetes mellitus), dyslipidemia [total cholesterol (TC) ≥5.2 mmol/L, triglycerides (TG) ≥1.7 mmol/L, low-density lipoprotein (LDL) ≥3.4 mmol/L, or high-density lipoprotein (HDL) < 1.0 mmol/L], or hyperuricemia (male ≥420 μmol/L, female ≥360 μmol/L); (3) first-time receipt of IL-17 inhibitor therapy (secukinumab or ixekizumab); (4) completion of at least 48 weeks of follow-up with available laboratory data at baseline and at least three post-baseline time points. Exclusion criteria comprised: (1) concurrent autoimmune diseases (rheumatoid arthritis, systemic lupus erythematosus, or inflammatory bowel disease); (2) prior exposure to any biologic agent; (3) severe hepatic or renal impairment [alanine aminotransferase (ALT) or aspartate aminotransferase (AST) > 3 × upper normal limit, estimated glomerular filtration rate (eGFR) < 60 mL/min/1.73 m^2^]; (4) pregnancy or lactation; (5) incomplete medical records or loss to follow-up.

### 2.3 Treatment protocol

All patients received IL-17 inhibitor therapy according to standard clinical protocols. Secukinumab (Cosentyx^®^, Novartis) was administered as 300 mg subcutaneous injection weekly for 5 weeks (weeks 0, 1, 2, 3, 4) followed by 300 mg every 4 weeks thereafter. Ixekizumab (Taltz^®^, Eli Lilly) was administered as 160 mg subcutaneous injection at week 0, followed by 80 mg every 2 weeks for 3 doses (weeks 2, 4, 6), then 80 mg every 4 weeks thereafter. Concomitant topical therapies (emollients, topical corticosteroids of mild to moderate potency) were permitted as needed for residual lesions, but no other systemic antipsoriatic treatments (including methotrexate, cyclosporine, acitretin, or other biologics) were allowed during the study period. Dose adjustments or treatment interruptions were recorded as part of safety monitoring.

### 2.4 Data collection

Two investigators extracted clinical and laboratory data from electronic medical records at baseline and weeks 12, 24, 36, 48. Discrepancies were resolved by consensus or a third investigator. Collected data included demographics (age, sex, BMI), metabolic comorbidities (obesity, dysglycemia, dyslipidemia, hyperuricemia, metabolic syndrome), psoriasis duration, baseline PASI score, prior treatment, and laboratory parameters from fasting blood samples (≥8 h). Concomitant medication data, including antidiabetic agents (e.g., metformin, sulfonylureas, DPP-4 inhibitors, SGLT2 inhibitors, insulin), lipid-lowering therapies (e.g., statins, fibrates, ezetimibe), and antihypertensive drugs, were also extracted at each visit to assess whether any dose adjustments or new initiations occurred during the 48-week follow-up period. However, lifestyle factors such as dietary intake, physical activity levels, smoking status, and alcohol consumption were not systematically recorded in the electronic medical records and could therefore not be included in the analysis.

### 2.5 Laboratory measurements

Fasting glucose was measured using the hexokinase method [coefficient of variation (CV) < 2%]. Lipid profiles including TC, TG, LDL, and HDL were measured by enzymatic methods using commercially available kits (Roche Diagnostics). LDL was calculated using the Friedewald formula when TG < 4.5 mmol/L. Uric acid was measured by the uricase-peroxidase method. Serum β2-microglobulin was measured by immunoturbidimetry (CV < 5%). UACR was calculated from spot urine samples using immunonephelometry for albumin and enzymatic method for creatinine. Serum homocysteine was measured by chemiluminescent microparticle immunoassay (Abbott Diagnostics). Complete blood counts including neutrophil, lymphocyte, monocyte, and platelet counts were performed using automated hematology analyzers (Sysmex XN-9000) with daily quality control.

### 2.6 Calculation of composite inflammatory indices

The following indices were calculated using baseline and follow-up complete blood count parameters: NLR = absolute neutrophil count/absolute lymphocyte count. PLR = platelet count/absolute lymphocyte count. SIRI = (neutrophil count × monocyte count)/lymphocyte count. SII = (neutrophil count × platelet count)/lymphocyte count. All calculations used absolute cell counts expressed as × 10^9^/L. For patients with multiple laboratory assessments within the same visit window, the value closest to the target date was used.

### 2.7 Outcome measures

The primary outcome was glycemic improvement defined as fasting glucose reduction ≥10% from baseline to week 48. This threshold was selected based on previous studies demonstrating that a 10% reduction in fasting glucose is associated with clinically meaningful improvements in insulin sensitivity and reduced risk of diabetes complications. Secondary outcomes included: (1) changes in lipid profiles (TC, TG, LDL, HDL) from baseline to each follow-up time point; (2) changes in uric acid, serum β2-microglobulin, UACR, and homocysteine; (3) changes in SIRI, SII, NLR, and PLR; (4) changes in PASI score; (5) PASI response rates (PASI 75, PASI 90, and PASI 100) at week 48; and (6) safety outcomes including documented adverse events during the treatment period. All outcomes were assessed using intention-to-treat principles with available data.

### 2.8 Statistical analysis

Statistical analyses used SPSS 26.0 and R 4.2.0. Normality was assessed via Shapiro-Wilk test. Normally distributed variables were mean ± SD (paired *t*-test); non-normal variables were median (IQR) (Wilcoxon signed-rank test). Categorical variables were n(%) (McNemar’s test). Correlations used Pearson or Spearman. Multivariate binary logistic regression (dependent: glycemic improvement, defined as ≥10% fasting glucose reduction at week 48) included variables with *P* < 0.10 in univariate analysis (age, sex, BMI, baseline PASI, baseline fasting glucose, SIRI reduction, SII reduction) via forward stepwise selection, reporting OR with 95% CI. Two-sided *P* < 0.05 was significant. No missing data imputation was performed.

## 3 Results

### 3.1 Patient disposition and baseline characteristics

**FIGURE 1:**
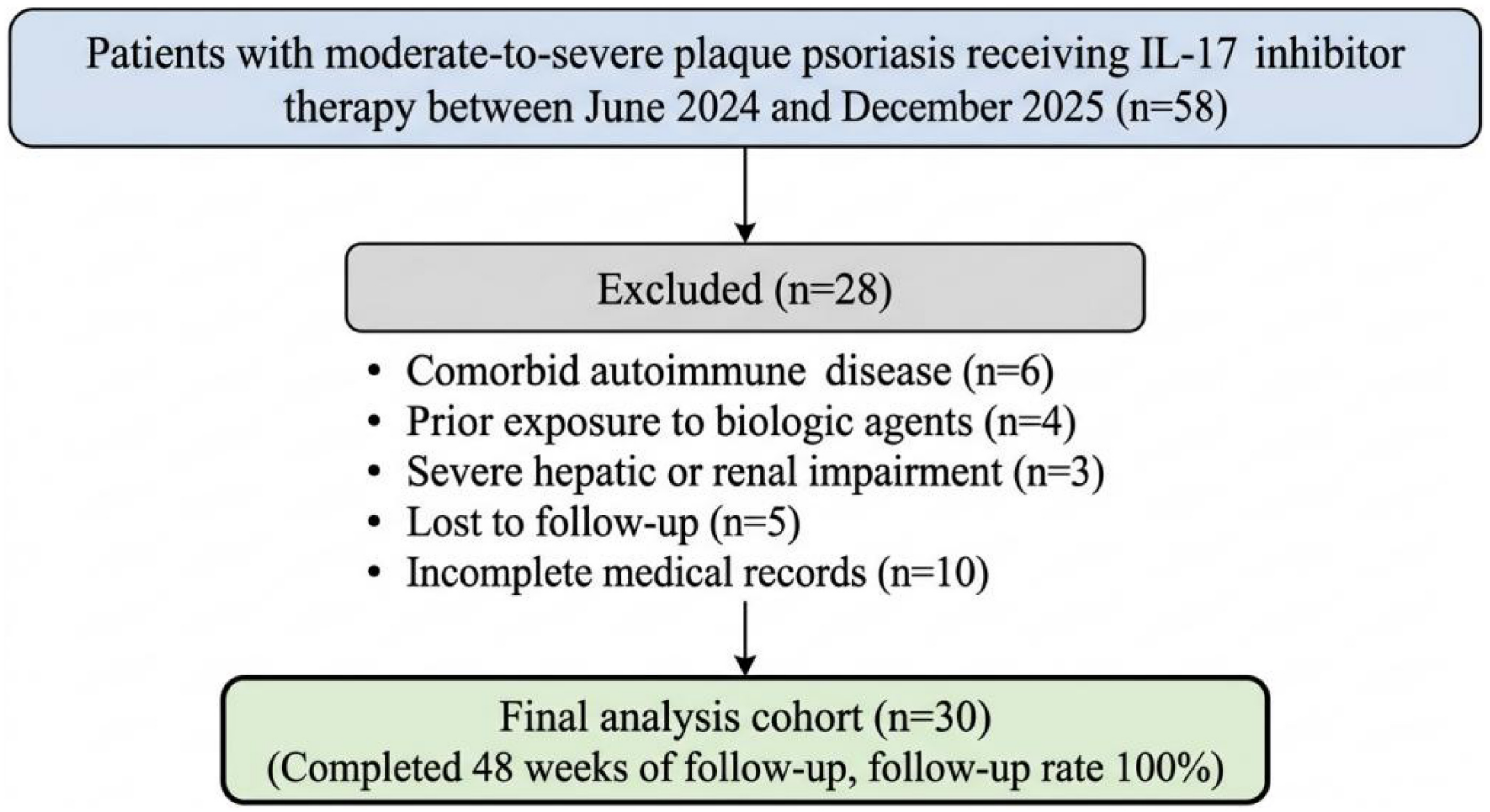
Of 58 screened patients with moderate-to-severe plaque psoriasis receiving IL-17 inhibitors (June 2024 to December 2025), 28 were excluded due to autoimmune diseases (*n* = 6), prior biologics (*n* = 4), severe hepatic/renal impairment (*n* = 3), loss to follow-up (*n* = 5), or incomplete data (*n* = 10). The remaining 30 patients completed 48 weeks of follow-up with data at all-time points (100% follow-up rate).

From June 2024 to December 2025, a total of 58 patients with moderate-to-severe plaque psoriasis who received IL-17 inhibitor therapy at XXX Unit were screened for eligibility. After applying inclusion and exclusion criteria, 28 patients were excluded: 6 due to concurrent autoimmune diseases, 4 due to prior biologic exposure, 3 due to severe hepatic or renal impairment, 5 lost to follow-up, and 10 with incomplete medical records. The final analytic cohort comprised 30 patients with moderate-to-severe plaque psoriasis and metabolic comorbidities, all of whom completed 48 weeks of clinical follow-up (follow-up rate 100%) (Figure 1).

Baseline characteristics of the 30 enrolled patients are presented in Table 1. The cohort comprised 18 male (60.0%) and 12 female (40.0%) patients, with mean age of 45.2 ± 10.3 years and mean BMI of 27.8 ± 3.5 kg/m^2^. Metabolic comorbidities included obesity in 16 patients (53.3%), dysglycemia in 14 (46.7%), dyslipidemia in 20 (66.7%), hyperuricemia in 10 (33.3%), and metabolic syndrome in 8 (26.7%). Mean psoriasis duration was 8.5 ± 4.2 years, and mean baseline PASI score was 18.6 ± 5.4, indicating severe psoriasis at treatment initiation. All patients had prior exposure to topical therapies, 12 (40.0%) had received phototherapy, and 8 (26.7%) had received conventional systemic agents (methotrexate or acitretin). Regarding IL-17 inhibitor type, 18 patients (60.0%) received secukinumab and 12 (40.0%) received ixekizumab, with no significant differences in baseline characteristics between the two treatment subgroups.

**TABLE 1:** Baseline demographic and clinical characteristics of the study population.

Characteristic	Total (*N* = 30)
Age, years, mean ± SD	45.2 ± 10.3
Male sex, *n* (%)	18 (60.0)
Female sex, *n* (%)	12 (40.0)
BMI, kg/m^2^, mean ± SD	27.8 ± 3.5
Psoriasis duration, years, mean ± SD	8.5 ± 4.2
Baseline PASI score, mean ± SD	18.6 ± 5.4
Metabolic comorbidities, n (%)	
Obesity (BMI ≥28 kg/m^2^)	16 (53.3)
Dysglycemia	14 (46.7)
Dyslipidemia	20 (66.7)
Hyperuricemia	10 (33.3)
Metabolic syndrome	8 (26.7)
Prior treatments, n (%)	
Topical therapy	30 (100)
Phototherapy	12 (40.0)
Conventional systemic agents	8 (26.7)
IL-17 inhibitor type, n (%)	
Secukinumab	18 (60.0)
Ixekizumab	12 (40.0)

Data are presented as mean ± SD or *n* (%). BMI, body mass index; PASI, Psoriasis Area and Severity Index; SD, standard deviation.

### 3.2 Changes in metabolic parameters

**FIGURE 2:**
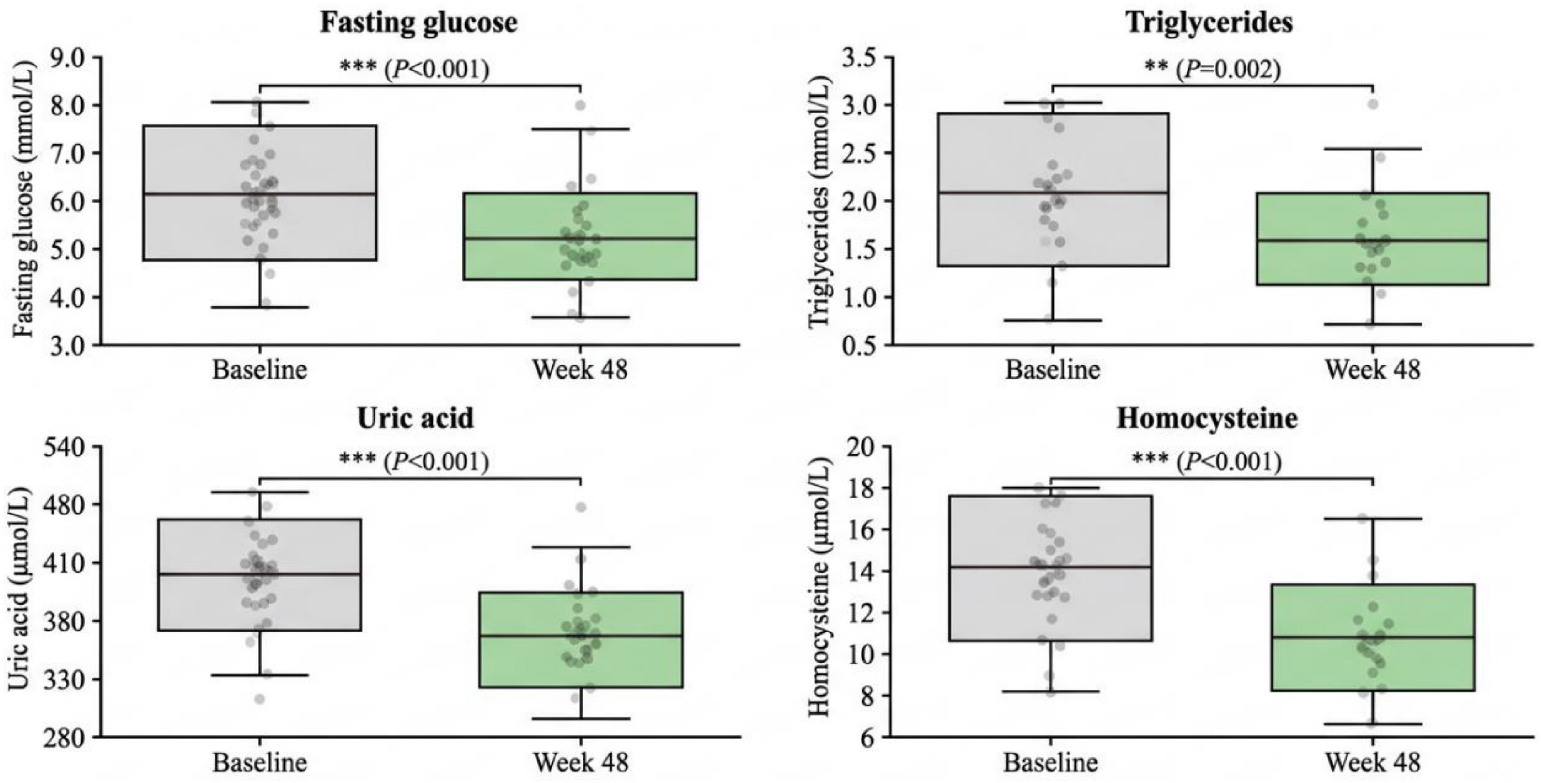
Box plots show baseline vs. week 48 decreases in fasting glucose (6.2 ± 1.4–5.3 ± 0.9 mmol/L, *P* < 0.001), triglycerides (2.1 ± 0.8–1.6 ± 0.5, *P* = 0.002), uric acid (435 ± 52–378 ± 41 μmol/L, *P* < 0.001), and homocysteine (14.2 ± 3.5–10.8 ± 2.6 μmol/L, *P* < 0.001). Box = IQR, line = median, whiskers = min/max, points = individual values. ***P < 0.001, **P < 0.01 (paired *t*-test).

Following 48 weeks of IL-17 inhibitor therapy, significant improvements were observed in multiple metabolic parameters (Figure 2 and Table 2). Fasting glucose decreased from 6.2 ± 1.4 mmol/L at baseline to 5.3 ± 0.9 mmol/L at week 48 (mean reduction 0.9 mmol/L, 95% CI: 0.5–1.3, *P* < 0.001). Among the 14 patients with baseline dysglycemia, 9 (64.3%) achieved normal fasting glucose (< 5.6 mmol/L) at week 48. TG decreased from 2.1 ± 0.8 mmol/L to 1.6 ± 0.5 mmol/L (mean reduction 0.5 mmol/L, 95% CI: 0.2–0.8, *P* = 0.002). Uric acid decreased from 435 ± 52 μmol/L to 378 ± 41 μmol/L (mean reduction 57 μmol/L, 95% CI: 35–79, *P* < 0.001). Homocysteine decreased from 14.2 ± 3.5 μmol/L to 10.8 ± 2.6 μmol/L (mean reduction 3.4 μmol/L, 95% CI: 2.1–4.7, P < 0.001). In contrast, TC (5.4 ± 1.1 vs. 5.2 ± 1.0 mmol/L, *P* = 0.245), LDL (3.2 ± 0.9 vs. 3.0 ± 0.8 mmol/L, *P* = 0.187), HDL (1.2 ± 0.3 vs. 1.3 ± 0.3 mmol/L, *P* = 0.089), serum β2-microglobulin (2.1 ± 0.6 vs. 1.9 ± 0.5 mg/L, *P* = 0.076), and UACR (18.5 ± 8.2 vs. 16.2 ± 7.5 mg/g, *P* = 0.124) showed no statistically significant changes. Notably, improvements in fasting glucose, TG, uric acid, and homocysteine were already evident at week 12 and were sustained through week 48, suggesting early and durable metabolic effects of IL-17 blockade (Table 2).

**TABLE 2:** Longitudinal changes in metabolic parameters during 48 weeks of IL-17 inhibitor therapy.

Parameter	Baseline (*n* = 30)	Week 12 (*n* = 30)	Week 24 (*n* = 30)	Week 36 (*n* = 30)	Week 48 (*n* = 30)	*P-*value*
Fasting glucose, mmol/L	6.2 ± 1.4	5.7 ± 1.1^†^	5.5 ± 1.0^†^	5.4 ± 0.9^†^	5.3 ± 0.9^†^	< 0.001
Total cholesterol, mmol/L	5.4 ± 1.1	5.3 ± 1.0	5.3 ± 1.0	5.2 ± 1.0	5.2 ± 1.0	0.245
Triglycerides, mmol/L	2.1 ± 0.8	1.8 ± 0.6^†^	1.7 ± 0.6^†^	1.6 ± 0.5^†^	1.6 ± 0.5^†^	0.002
LDL, mmol/L	3.2 ± 0.9	3.1 ± 0.8	3.1 ± 0.8	3.0 ± 0.8	3.0 ± 0.8	0.187
HDL, mmol/L	1.2 ± 0.3	1.2 ± 0.3	1.3 ± 0.3	1.3 ± 0.3	1.3 ± 0.3	0.089
Uric acid, μmol/L	435 ± 52	402 ± 45^†^	389 ± 43^†^	382 ± 42^†^	378 ± 41^†^	< 0.001
β2-microglobulin, mg/L	2.1 ± 0.6	2.0 ± 0.6	2.0 ± 0.5	1.9 ± 0.5	1.9 ± 0.5	0.076
UACR, mg/g	18.5 ± 8.2	17.8 ± 7.9	17.1 ± 7.8	16.5 ± 7.6	16.2 ± 7.5	0.124
Homocysteine, μmol/L	14.2 ± 3.5	12.1 ± 3.0^†^	11.4 ± 2.8^†^	11.0 ± 2.7^†^	10.8 ± 2.6^†^	< 0.001

Data are presented as mean ± SD. *P-values represent comparison between baseline and week 48 using paired *t-*tests.

† *P* < 0.05 versus baseline by paired *t*-test with Bonferroni correction for multiple comparisons. LDL, low-density lipoprotein; HDL, high-density lipoprotein; UACR, urinary albumin-to-creatinine ratio.

### 3.3 Changes in inflammatory indices and PASI score

**FIGURE 3:**
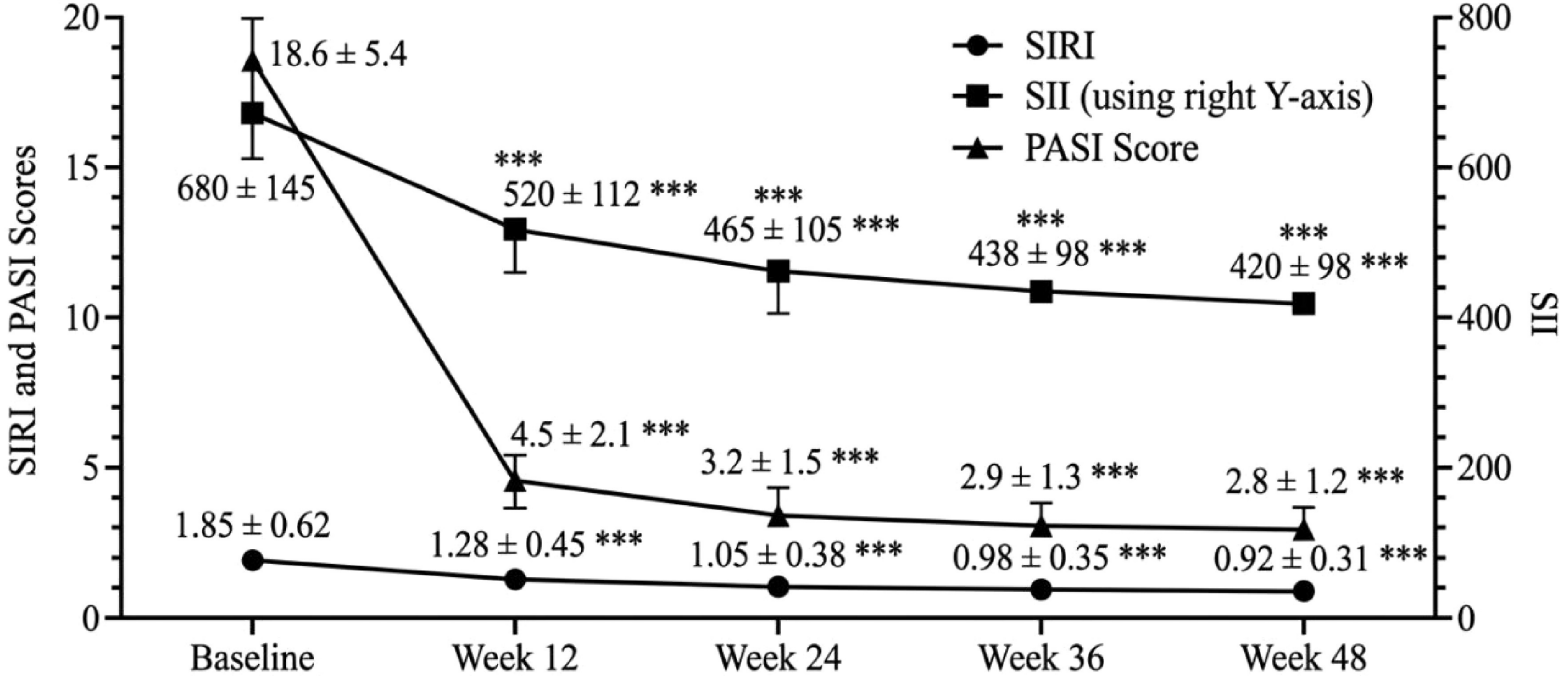
Line graphs show 48-week decreases in SIRI (1.85 ± 0.62 baseline to 0.92 ± 0.31 at week 48), SII (680 ± 145–420 ± 98), and PASI (18.6 ± 5.4–2.8 ± 1.2), with reductions evident by week 12 and sustained through week 48 (*P* < 0.001 for all post-baseline time points vs. baseline, paired *t*-test with Bonferroni correction). Data points = means; error bars = SD. ***P < 0.001.

Marked reductions in systemic inflammatory indices were observed throughout the 48-week treatment period (Figure 3 and Table 3). SIRI decreased progressively from baseline (1.85 ± 0.62) to week 12 (1.28 ± 0.45, *P* < 0.001), week 24 (1.05 ± 0.38, *P* < 0.001), week 36 (0.98 ± 0.35, *P* < 0.001), and week 48 (0.92 ± 0.31, *P* < 0.001), representing a mean reduction of 50.3% from baseline. Similarly, SII decreased from 680 ± 145 at baseline to 520 ± 112 at week 12 (*P* < 0.001), 465 ± 105 at week 24 (*P* < 0.001), 438 ± 98 at week 36 (*P* < 0.001), and 420 ± 98 at week 48 (*P* < 0.001), representing a mean reduction of 38.2%. PASI score demonstrated rapid improvement from 18.6 ± 5.4 at baseline to 4.5 ± 2.1 at week 12 (*P* < 0.001), with continued gradual improvement to 2.8 ± 1.2 at week 48 (*P* < 0.001). At week 48, PASI 75 response was achieved in 28 patients (93.3%), PASI 90 response in 21 patients (70.0%), and PASI 100 response in 11 patients (36.7%). Individual components of the inflammatory indices showed consistent improvements: neutrophil counts decreased from 4.85 ± 1.20 × 10^9^/L to 3.48 ± 0.78 × 10^9^/L (*P* < 0.001), monocyte counts decreased from 0.58 ± 0.18 × 10^9^/L to 0.43 ± 0.12 × 10^9^/L (*P* < 0.001), and platelet counts decreased from 268 ± 65 × 10^9^/L to 232 ± 50 × 10^9^/L (*P* = 0.003). Lymphocyte counts showed a modest but non-significant increase from 1.95 ± 0.55 × 10^9^/L to 2.15 ± 0.46 × 10^9^/L (*P* = 0.087). Both NLR and PLR also demonstrated significant reductions (both *P* < 0.01), consistent with the overall attenuation of systemic inflammation.

**TABLE 3:** Longitudinal changes in inflammatory indices and PASI score during 48 weeks of IL-17 inhibitor therapy.

Parameter	Baseline (*n* = 30)	Week 12 (*n* = 30)	Week 24 (*n* = 30)	Week 36 (*n* = 30)	Week 48 (*n* = 30)	*P*-value*
Neutrophils, × 10^9^/L	4.85 ± 1.20	3.95 ± 0.98^†^	3.65 ± 0.85^†^	3.52 ± 0.80^†^	3.48 ± 0.78^†^	< 0.001
Lymphocytes, × 10^9^/L	1.95 ± 0.55	2.05 ± 0.52	2.10 ± 0.50	2.12 ± 0.48	2.15 ± 0.46	0.087
Monocytes, × 10^9^/L	0.58 ± 0.18	0.48 ± 0.15^†^	0.45 ± 0.14^†^	0.44 ± 0.13^†^	0.43 ± 0.12^†^	< 0.001
Platelets, × 10^9^/L	268 ± 65	245 ± 58^†^	238 ± 55^†^	235 ± 52^†^	232 ± 50^†^	0.003
NLR	2.52 ± 0.95	1.96 ± 0.72^†^	1.78 ± 0.65^†^	1.70 ± 0.60^†^	1.65 ± 0.58^†^	< 0.001
PLR	140.2 ± 45.5	121.5 ± 38.2^†^	115.8 ± 35.6^†^	112.4 ± 34.1^†^	110.2 ± 33.5^†^	0.002
SIRI	1.85 ± 0.62	1.28 ± 0.45^†^	1.05 ± 0.38^†^	0.98 ± 0.35^†^	0.92 ± 0.31^†^	< 0.001
SII	680 ± 145	520 ± 112^†^	465 ± 105^†^	438 ± 98^†^	420 ± 98^†^	< 0.001
PASI score	18.6 ± 5.4	4.5 ± 2.1^†^	3.2 ± 1.5^†^	2.9 ± 1.3^†^	2.8 ± 1.2^†^	< 0.001

Data are presented as mean ± SD. *P-values represent comparison between baseline and week 48 using paired *t*-tests.

† *P* < 0.05 versus baseline by paired *t*-test with Bonferroni correction for multiple comparisons. NLR, neutrophil-to-lymphocyte ratio; PLR, platelet-to-lymphocyte ratio; SIRI, systemic inflammation response index; SII, systemic immune-inflammation index; PASI, Psoriasis Area and Severity Index.

### 3.4 Correlation analyses

**FIGURE 4:**
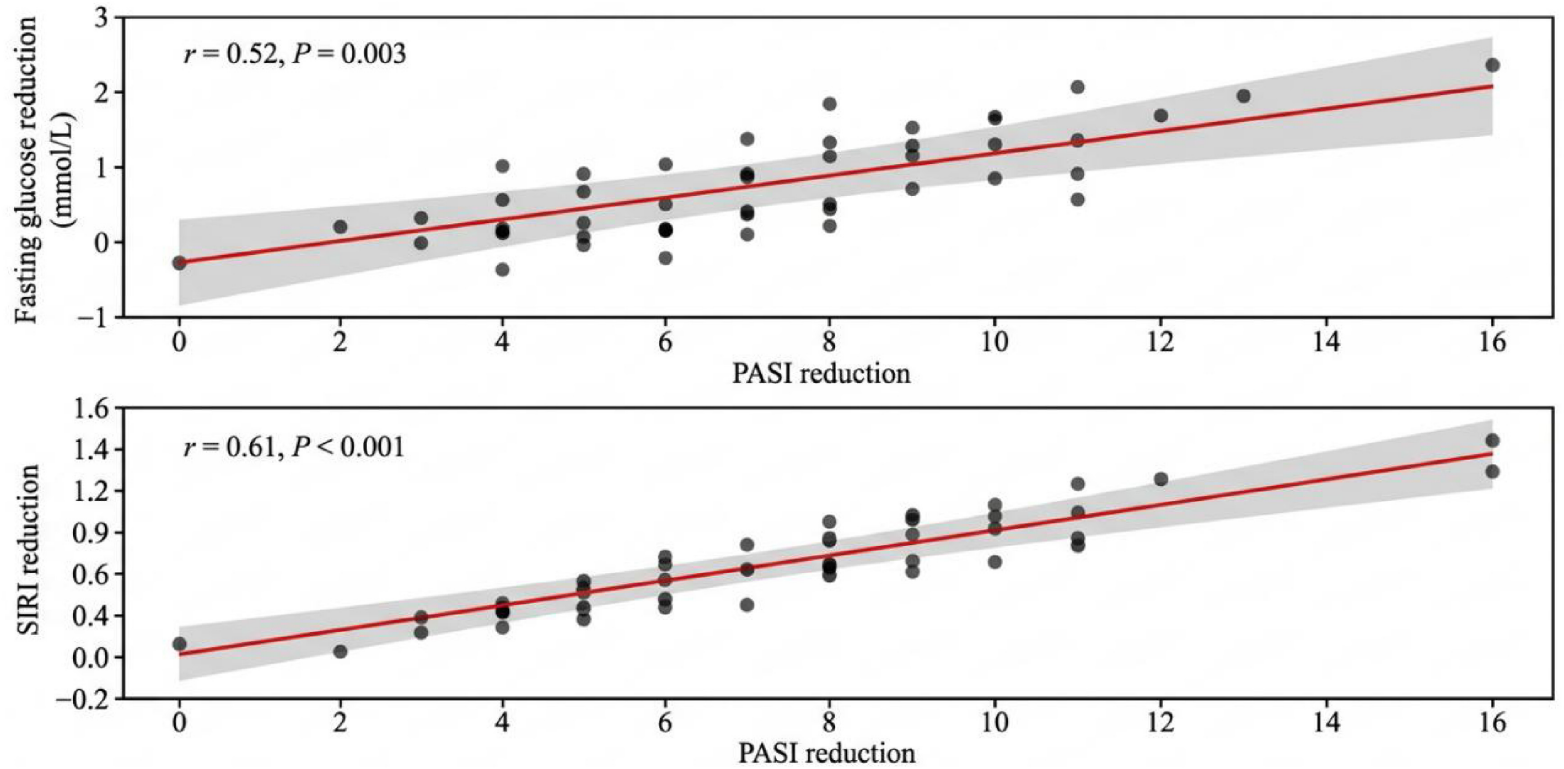
Scatter plots show positive correlations at week 48 between PASI reduction and fasting glucose reduction (*r* = 0.52, *P* = 0.003) and between PASI reduction and SIRI reduction (*r* = 0.61, *P* < 0.001). The stronger correlation with SIRI suggests that reduced systemic inflammation may mediate metabolic improvements with IL-17 inhibitor therapy. Lines = linear regression; shaded area = 95% CI.

Correlation analyses revealed significant positive associations between PASI score reduction and improvements in metabolic parameters and inflammatory indices at week 48 (Figure 4 and Table 4). The reduction in PASI score was positively correlated with reduction in fasting glucose (*r* = 0.52, *P* = 0.003), reduction in TG (*r* = 0.45, *P* = 0.012), reduction in uric acid (*r* = 0.48, *P* = 0.007), and reduction in homocysteine (*r* = 0.43, *P* = 0.018). Notably, stronger correlations were observed between PASI reduction and reductions in SIRI (*r* = 0.61, *P* < 0.001) and SII (*r* = 0.55, *P* = 0.002), suggesting that attenuation of systemic inflammation may mediate the metabolic improvements associated with IL-17 inhibitor therapy. In contrast, changes in TC, LDL, β2-microglobulin, and UACR showed no significant correlations with PASI reduction (all *P* > 0.10). When analyses were stratified by baseline metabolic status, the correlations between PASI reduction and metabolic improvements were numerically stronger in patients with dysglycemia at baseline (*r* = 0.65 for fasting glucose reduction) compared with those without (*r* = 0.38), suggesting that the metabolic benefits may be more pronounced in patients with pre-existing metabolic abnormalities.

**TABLE 4:** Correlation analysis between PASI score reduction and changes in metabolic and inflammatory parameters at week 48.

Parameter change (week 48—baseline)	Correlation coefficient (r)	*P-*value
Fasting glucose reduction	0.52	0.003
Triglycerides reduction	0.45	0.012
Uric acid reduction	0.48	0.007
Homocysteine reduction	0.43	0.018
SIRI reduction	0.61	< 0.001
SII reduction	0.55	0.002
Total cholesterol reduction	0.28	0.132
LDL reduction	0.25	0.185
β2-microglobulin reduction	0.22	0.245
UACR reduction	0.19	0.312
NLR reduction	0.53	0.003
PLR reduction	0.48	0.008

Pearson correlation coefficients (r) are reported for normally distributed variables. PASI, Psoriasis Area and Severity Index; SIRI, systemic inflammation response index; SII, systemic immune-inflammation index; LDL, low-density lipoprotein; UACR, urinary albumin-to-creatinine ratio; NLR, neutrophil-to-lymphocyte ratio; PLR, platelet-to-lymphocyte ratio.

### 3.5 Multivariate analysis of factors associated with glycemic improvement

Binary logistic regression analysis was performed to identify independent predictors of glycemic improvement (defined as fasting glucose reduction ≥10% at week 48) (Table 5). In univariate analysis, baseline fasting glucose (OR = 1.92, 95% CI: 1.15–3.21, *P* = 0.013), SIRI reduction (OR = 2.85, 95% CI: 1.32–6.15, *P* = 0.008), and SII reduction (OR = 2.31, 95% CI: 1.08–4.94, *P* = 0.031) were significantly associated with glycemic improvement. Age, sex, BMI, baseline PASI score, and psoriasis duration showed no significant associations. Multivariate analysis adjusting for age, sex, BMI, baseline PASI score, and baseline fasting glucose identified SIRI reduction as an independent predictor of glycemic improvement (OR = 2.68, 95% CI: 1.21–5.93, *P* = 0.015). SII reduction also demonstrated independent predictive value (OR = 2.12, 95% CI: 1.03–4.38, *P* = 0.042). Baseline fasting glucose remained an independent predictor (OR = 1.85, 95% CI: 1.12–3.06, *P* = 0.016). The area under the receiver operating characteristic curve for the model including SIRI reduction and baseline fasting glucose was 0.81 (95% CI: 0.72–0.90), indicating good discriminative ability for identifying patients likely to achieve glycemic improvement.

**TABLE 5:** Univariate and multivariate logistic regression analyses for predictors of glycemic improvement (fasting glucose reduction ≥10% at week 48).

Variable	Univariate analysis	Multivariate analysis		
Project	OR (95% CI)	*P-*value	OR (95% CI)	*P-*value
Age (per year)	1.02 (0.97–1.07)	0.452	1.01 (0.96–1.06)	0.685
Sex (male vs. female)	1.35 (0.68–2.68)	0.392	1.28 (0.62–2.64)	0.502
BMI (per kg/m^2^)	1.08 (0.94–1.24)	0.285	1.05 (0.91–1.21)	0.498
Baseline PASI score (per point)	1.04 (0.96–1.13)	0.342	1.02 (0.94–1.11)	0.615
Baseline fasting glucose (per mmol/L)	1.92 (1.15–3.21)	0.013	1.85 (1.12–3.06)	0.016
SIRI reduction (per 0.5 unit)	2.85 (1.32–6.15)	0.008	2.68 (1.21–5.93)	0.015
SII reduction (per 100 units)	2.31 (1.08–4.94)	0.031	2.12 (1.03–4.38)	0.042

OR, odds ratio; CI, confidence interval; BMI, body mass index; PASI, Psoriasis Area and Severity Index; SIRI, systemic inflammation response index; SII, systemic immune-inflammation index. Multivariate analysis adjusted for age, sex, BMI, baseline PASI score, and baseline fasting glucose using forward stepwise selection. Variables with *P* < 0.10 in univariate analysis were entered into the multivariate model.

### 3.6 Safety outcomes

During the 48-week treatment period, no serious adverse events were reported. Mild injection site reactions (erythema, pain, or swelling at the injection site) occurred in 4 patients (13.3%), all of which resolved spontaneously within 24–48 h without intervention. Upper respiratory tract infections were reported in 3 patients (10.0%), all of mild severity and none requiring treatment discontinuation or hospitalization. No cases of candidiasis, inflammatory bowel disease, or major adverse cardiovascular events were observed. No patients discontinued treatment due to adverse events. Laboratory monitoring revealed no clinically significant abnormalities in liver or renal function tests throughout the study period.

### 3.7 Concomitant medication use

During the 48-week follow-up period, we reviewed concomitant medication records for all 30 patients. Regarding antidiabetic agents, among the 14 patients with baseline dysglycemia, 6 were on stable doses of metformin, 2 on sulfonylureas, and 1 on a DPP-4 inhibitor; no dose adjustments or new initiations of antidiabetic medications were recorded during the study period. For lipid-lowering therapy, among the 20 patients with baseline dyslipidemia, 8 were receiving statins (atorvastatin or rosuvastatin) at stable doses, and 2 were on fenofibrate; no dose changes or treatment initiations occurred during follow-up. No patients were started on antihypertensive agents during the study. Thus, the observed metabolic improvements are unlikely to be attributable to changes in concomitant metabolic medications, although we cannot completely exclude the influence of unmeasured lifestyle modifications.

## 4 Discussion

The core innovation of this research lies in three key aspects that collectively address critical gaps in the existing literature. First, this study provides the first comprehensive longitudinal assessment of multiple metabolic parameters (fasting glucose, TG, uric acid, homocysteine) over 48 weeks in psoriasis patients with metabolic comorbidities treated with IL-17 inhibitors, extending the typical 12–24 week follow-up durations in previous studies. Second, this is the first study to systematically characterize the dynamic changes of novel composite inflammatory indices SIRI and SII during IL-17 inhibitor therapy and to demonstrate their independent predictive value for glycemic improvement. Third, our finding that SIRI reduction was associated with glycemic improvement (OR = 2.68, *P* = 0.015) suggests a potential mechanistic link between inflammation attenuation and metabolic benefit, offering preliminary rationale for considering SIRI as a candidate clinical biomarker for monitoring treatment response in future studies. The strong correlation between PASI reduction and SIRI reduction (*r* = 0.61, *P* < 0.001) further supports the concept that systemic inflammation drives both cutaneous and metabolic manifestations of psoriatic disease, and that effective IL-17 blockade yields pleiotropic benefits beyond skin clearance.

Our results align with and extend several recent high-impact investigations examining metabolic effects of IL-17 inhibitors in psoriasis. A prospective study demonstrated that secukinumab treatment for 52 weeks reduced fasting glucose by 0.6 mmol/L and improved homeostatic model assessment for insulin resistance (HOMA-IR) by 28% in psoriasis patients with prediabetes, consistent with our observed 0.9 mmol/L reduction (14.5% decrease) and the 64.3% rate of reversion to normoglycemia among dysglycemic patients (14). Similarly, a Danish nationwide registry study reported that IL-17 inhibitor users had a 32% lower risk of incident type 2 diabetes compared with non-biologic users after adjusting for baseline risk factors, suggesting that the glycemic benefits observed in clinical trials translate into real-world risk reduction (15). Regarding lipid profiles, our finding of TG reduction without significant changes in TC or LDL aligns with a recent network meta-analysis showing that biologic therapies preferentially affect TG levels rather than other lipid fractions, possibly due to the distinct effects of inflammatory cytokines on very-low-density lipoprotein metabolism and lipoprotein lipase activity (16). The novel observation of uric acid reduction (57 μmol/L, 13.1% decrease) extends preliminary findings from a prospective cohort study that reported ixekizumab treatment reduced serum uric acid levels by approximately 10% in psoriasis patients with hyperuricemia (17). This is the first study reporting significant homocysteine reduction with IL-17 inhibitor therapy, which may have implications for cardiovascular risk modification, as elevated homocysteine is an independent risk factor for cardiovascular events, stroke, and venous thromboembolism (18).

The mechanistic basis for the observed metabolic improvements likely involves multiple interconnected pathways downstream of IL-17 receptor signaling. IL-17 is a critical driver of adipose tissue inflammation, where it promotes recruitment of pro-inflammatory M1 macrophages and induces adipocyte secretion of tumor necrosis factor (TNF)-α, IL-6, and resistin, leading to impaired insulin signaling through serine phosphorylation of insulin receptor substrate-1 (19). Blockade of IL-17 signaling reduces adipose tissue macrophage infiltration, shifts macrophage polarization toward the anti-inflammatory M2 phenotype, and restores insulin sensitivity in both adipocytes and hepatocytes. IL-17 directly modulates hepatic lipid metabolism by upregulating sterol regulatory element-binding protein 1c (SREBP-1c) expression, a master transcriptional regulator of lipogenesis, thereby promoting de novo lipogenesis and very-low-density lipoprotein secretion (20). IL-17 inhibitors may reduce hepatic TG synthesis and secretion, explaining the preferential reduction in TG levels observed in our study. IL-17 promotes uric acid production by enhancing xanthine oxidase activity in the liver and reducing renal uric acid excretion through downregulation of urate transporter 1 (URAT1) (21). IL-17-induced oxidative stress increases homocysteine production by upregulating methionine metabolism enzymes, while IL-17 blockade reduces homocysteine levels through attenuation of oxidative stress and preservation of renal function. The strong correlation between SIRI reduction and metabolic improvements in our study supports the central role of systemic inflammation attenuation in mediating these effects, consistent with the emerging paradigm that targeting inflammation can improve metabolic outcomes across multiple disease contexts (22).

The clinical implications of our findings are substantial and practice-relevant. For the approximately 30–40% of psoriasis patients who have concurrent metabolic comorbidities, IL-17 inhibitors may offer dual benefits: highly effective skin clearance plus meaningful improvements in glycemic control, TG levels, uric acid, and homocysteine (23). However, it remains unclear whether the observed metabolic improvements are specifically attributable to IL-17 blockade or rather reflect a class effect of effective biologic therapy through systemic inflammation reduction. Since our study lacked a comparator group receiving other biologic classes (e.g., TNF inhibitors, IL-23 inhibitors, or IL-12/23 inhibitors), we cannot determine whether IL-17 inhibitors confer superior metabolic benefits compared with other biologics. Therefore, while our findings are encouraging, the suggestion that IL-17 inhibitors may represent a preferred therapeutic option for patients with type 2 diabetes mellitus or other metabolic comorbidities remains speculative at this stage and requires confirmation through head-to-head comparative trials. If confirmed in larger prospective studies, this would position IL-17 inhibitors as particularly attractive options for patients with psoriasis and type 2 diabetes, hypertriglyceridemia, or hyperuricemia. Preliminarily, the SIRI, derived from routine complete blood counts, could potentially represent a practical, low-cost biomarker that can be easily calculated in any clinical setting without additional laboratory costs. Our finding that SIRI reduction was associated with glycemic improvement raises the hypothesis that monitoring SIRI during treatment might help identify patients likely to achieve metabolic benefits, although this requires prospective validation. However, we acknowledge that the practical utility of SIRI and SII as markers of concurrent clinical response is limited, given that psoriasis improvement is readily assessable through standard clinical tools such as PASI and physician global assessment. A potentially more valuable application, which our current study was not designed to address, would be to investigate whether these indices could serve as predictive biomarkers of future loss of efficacy. For instance, a progressive increase in SIRI or SII during maintenance therapy might precede clinical relapse or worsening of psoriasis, enabling early intervention before overt disease flare. This hypothesis warrants investigation in future longitudinal studies with extended follow-up and frequent biomarker monitoring between clinical visits. Pending confirmation from future studies, a hypothesis-generating algorithm could be proposed for research purposes: calculating SIRI at baseline and at 12–24 weeks; patients with substantial SIRI reduction (> 50%) might hypothetically have favorable metabolic responses, whereas those with minimal SIRI reduction might potentially require closer monitoring of metabolic parameters or consideration of alternative treatment strategies. However, such an algorithm should not be implemented clinically without prospective validation. Furthermore, the observed homocysteine reduction raises the hypothesis that IL-17 inhibitors might reduce cardiovascular risk in psoriasis patients, although this requires confirmation in dedicated long-term cardiovascular outcome trials. Given that recent large-scale trials have demonstrated that inflammation reduction lowers cardiovascular event rates independent of lipid lowering, it is plausible that IL-17 inhibition may have similar effects, warranting further investigation (24).

Several biological plausibility considerations strengthen confidence in our findings. Psoriasis and metabolic syndrome share the IL-23/Th17 inflammatory axis as a common pathogenic driver, providing a unifying mechanism for the observed association between skin disease activity and metabolic disturbances (25). The temporal sequence in our data—improvements in SIRI and SII preceding or coinciding with metabolic improvements—supports a causal relationship rather than mere association. The dose-response-like pattern, with greater PASI reduction correlating with greater metabolic improvement (*r* = 0.52–0.61), further supports this interpretation. Additionally, the fact that improvements were observed across multiple metabolically relevant parameters (glucose, TG, uric acid, homocysteine) rather than a single isolated parameter argues against chance findings. The early onset of metabolic improvements (evident by week 12) and their durability through week 48 suggest that these effects are sustained with continued treatment, which is clinically important given the chronic nature of both psoriasis and metabolic diseases. The consistency of our findings with preclinical mechanistic studies and with clinical trials of other inflammatory conditions provides additional convergent validity (26).

Despite the strengths of this study, several limitations must be acknowledged. First, the retrospective single-center design introduces potential selection bias, as treatment decisions were not randomized and unmeasured confounding factors may have influenced the observed associations. Although we adjusted for key confounders (age, sex, BMI, baseline PASI, baseline fasting glucose) in multivariate analysis, we cannot exclude the possibility of residual confounding by factors such as diet, physical activity, concomitant medications (e.g., metformin, statins, antihypertensives), or treatment adherence. We did systematically extract concomitant medication data at each visit and confirmed that no dose adjustments or new initiations of antidiabetic or lipid-lowering agents occurred during the study period (see Results 3.7). Nevertheless, we acknowledge that we did not collect data on lifestyle factors (dietary patterns, physical activity levels, smoking, or alcohol consumption), which could have influenced metabolic parameters independently of treatment. The absence of these data represents a significant barrier to establishing causality. Importantly, the absence of a control group precludes definitive attribution of the observed metabolic improvements specifically to IL-17 inhibitor therapy, as we cannot rule out the potential contributions of natural disease course fluctuations, spontaneous regression to the mean, concurrent lifestyle modifications (e.g., dietary changes or increased physical activity initiated after treatment commencement), or the effects of concomitant medications that may have been adjusted during the follow-up period. Without a comparator group receiving conventional systemic therapy, placebo, or no biologic treatment, the observed improvements in glycemic and lipid parameters could partially reflect background variability rather than true treatment effects. Second, the relatively small sample size (*n* = 30) limits statistical power for subgroup analyses and precludes definitive conclusions about differential effects between secukinumab and ixekizumab or across different metabolic comorbidity profiles. The sample size was constrained by the 18-month data collection window (June 2023 to December 2024) and the stringent eligibility criteria requiring concurrent metabolic comorbidities, complete 48-week follow-up data, and at least three post-baseline laboratory time points. These criteria, while necessary to ensure data quality and outcome reliability, inevitably reduced the number of eligible patients. Furthermore, patients were distributed between two IL-17 inhibitors (secukinumab, *n* = 18; ixekizumab, *n* = 12), which further limited our ability to perform meaningful comparisons between treatment subtypes. The 95% confidence intervals for some effect estimates were relatively wide, indicating imprecision. *Post-hoc* power analysis confirmed 80% power for the primary outcome, but subgroup analyses were underpowered. Consequently, the possibility of type II errors for secondary outcomes cannot be excluded, and the generalizability of our findings to broader psoriasis populations or other clinical settings remains uncertain. Future studies with extended recruitment periods and multi-center collaboration are warranted to achieve larger sample sizes that would enable robust subgroup analyses. Third, the absence of a control group (e.g., patients receiving conventional systemic therapy or placebo) means we cannot definitively attribute the observed metabolic improvements to IL-17 inhibitor therapy rather than to regression to the mean, spontaneous improvement, or concurrent lifestyle modifications. This limitation is particularly pertinent given that metabolic parameters such as fasting glucose and triglycerides can exhibit substantial within-individual variability over time independent of therapeutic intervention. Fourth, we did not assess insulin resistance directly using gold-standard methods such as hyperinsulinemic-euglycemic clamps or the frequently sampled intravenous glucose tolerance test. HOMA-IR, which we plan to include in future analyses, provides only an indirect estimate of insulin sensitivity and is influenced by both hepatic and peripheral insulin resistance. The absence of direct measures of insulin resistance further limits our ability to establish a causal link between IL-17 inhibition and improved glycemic control. Fifth, we did not systematically collect data on lifestyle factors (diet, physical activity, smoking, alcohol consumption) or concurrent medication changes (e.g., initiation or dose adjustment of antidiabetic or lipid-lowering agents) that could have confounded the observed associations. Additionally, we did not assess insulin resistance directly using gold-standard methods such as hyperinsulinemic-euglycemic clamps; HOMA-IR, which we plan to include in future analyses, provides only an indirect estimate. The 48-week follow-up, while longer than many previous studies, is insufficient to determine whether the observed metabolic improvements translate into reduced risks of hard clinical endpoints such as myocardial infarction, stroke, or new-onset diabetes. Given these limitations, the findings of this study should be considered hypothesis-generating rather than definitive. The observed associations, while encouraging, require confirmation in well-designed prospective cohorts or randomized controlled trials before any clinical recommendations can be made regarding the metabolic benefits of IL-17 inhibitors or the utility of SIRI as a clinical biomarker. Readers are cautioned against overinterpreting the results as proof of causality or as sufficient evidence to guide clinical decision-making in the absence of confirmatory studies.

Future research directions should address these limitations through several approaches. Multi-center prospective cohort studies with larger sample sizes (target *n* > 200) are needed to validate our findings and enable robust subgroup analyses examining effect modification by baseline characteristics (age, sex, BMI, metabolic comorbidity type) and treatment type (secukinumab versus ixekizumab versus other IL-17 inhibitors). Most critically, head-to-head randomized controlled trials comparing IL-17 inhibitors with other biologic classes (TNF inhibitors, IL-23 inhibitors, IL-12/23 inhibitors) are urgently needed to determine whether the observed metabolic improvements are specific to IL-17 blockade or represent a shared class effect of biologic therapy. Such comparative studies would clarify whether IL-17 inhibitors offer distinct advantages over other biologics in patients with metabolic comorbidities or whether the benefits are broadly attributable to systemic inflammation reduction irrespective of the therapeutic target. Such trials should include rigorous assessment of insulin sensitivity (using HOMA-IR or, in subsets, hyperinsulinemic-euglycemic clamps), comprehensive lipid profiling (including apolipoprotein B, lipoprotein(a), and lipid particle size), and measurement of additional inflammatory biomarkers (IL-6, TNF-α, high-sensitivity CRP). Such trials should include rigorous assessment of insulin sensitivity (using HOMA-IR or, in subsets, hyperinsulinemic-euglycemic clamps), comprehensive lipid profiling (including apolipoprotein B, lipoprotein(a), and lipid particle size), and measurement of additional inflammatory biomarkers (IL-6, TNF-α, high-sensitivity CRP). Long-term follow-up studies extending to 3–5 years are essential to determine whether the observed improvements in intermediate metabolic endpoints translate into reduced incidence of type 2 diabetes, major adverse cardiovascular events, and chronic kidney disease. Investigation of the molecular mechanisms underlying IL-17-mediated metabolic dysregulation using preclinical models would provide mechanistic insights complementing clinical observations. Finally, cost-effectiveness analyses are warranted to determine whether the additional metabolic benefits of IL-17 inhibitors justify their higher cost compared with conventional systemic therapies in patients with metabolic comorbidities. Given the increasing recognition of the link between inflammation and metabolic disease, our findings may have implications beyond psoriasis, suggesting that IL-17 inhibition could be a therapeutic strategy for metabolic diseases even in the absence of psoriasis.

## 5 Conclusion

In conclusion, this retrospective cohort study suggests that 48 weeks of IL-17 inhibitor therapy in patients with moderate-to-severe plaque psoriasis and metabolic comorbidities is associated with improvements in glycemic control, TG, uric acid, and homocysteine, accompanied by notable reductions in systemic inflammatory indices SIRI and SII. Exploratory analyses indicated that SIRI reduction was associated with glycemic improvement in this cohort, raising the hypothesis of a potential mechanistic link between inflammation attenuation and metabolic benefit. However, due to the observational design, the absence of a control group, the lack of systematic lifestyle data, and the absence of direct measures of insulin resistance, we cannot establish a causal relationship between IL-17 inhibition and the observed metabolic improvements. The possibility that unmeasured confounding factors (e.g., dietary changes, physical activity) contributed to these findings cannot be excluded. While SIRI and SII reductions paralleled PASI improvement, their incremental value as markers of concurrent clinical response is limited given that psoriasis improvement is readily assessed by standard clinical tools. A more promising avenue for future research-which our current study was not powered to explore-would be to investigate whether increases in these indices during maintenance therapy could serve as early predictive signals of impending loss of efficacy or clinical relapse. Pending prospective validation, the SIRI, calculated from routine complete blood counts, may represent a candidate biomarker for monitoring treatment response in future research settings. If confirmed in rigorously designed prospective controlled trials, these preliminary findings would suggest that IL-17 inhibitors could be considered as treatment options for psoriasis patients with concurrent metabolic abnormalities, potentially offering dual benefits of effective skin clearance and favorable metabolic improvements. However, given the observational design, small sample size, and absence of a control group, these findings should be interpreted as hypothesis-generating rather than conclusive. Future large-scale, multi-center prospective controlled studies with larger sample sizes and longer follow-up are warranted to validate these findings and to establish causality before any clinical recommendations can be made.

## Data Availability

The original contributions presented in the study are included in the article/supplementary material, further inquiries can be directed to the corresponding author.
